# The role of dynamic, static, and delayed total-body PET imaging in the detection and differential diagnosis of oncological lesions

**DOI:** 10.1186/s40644-023-00649-5

**Published:** 2024-01-02

**Authors:** Yaping Wu, Fangfang Fu, Nan Meng, Zhenguo Wang, Xiaochen Li, Yan Bai, Yun Zhou, Dong Liang, Hairong Zheng, Yongfeng Yang, Meiyun Wang, Tao Sun

**Affiliations:** 1 Department of Medical Imaging, Henan Provincial People’s Hospital and the People’s Hospital of Zhengzhou, University of Zhengzhou, Zhengzhou, Henan People’s Republic of China; 2 Paul C. Lauterbur Research Center for Biomedical Imaging, Shenzhen Institute of Advanced Technology, Chinese Academy of Sciences, Shenzhen, Guangdong People’s Republic of China; 3 School of Biomedical Engineering, Shanghai Tech University, Shanghai, People’s Republic of China; 4 Laboratory of Brain Science and Brain-Like Intelligence TechnologyInstitute for Integrated Medical Science and Engineering, Henan Academy of Sciences, Zhengzhou, Henan People’s Republic of China; 5 Research Institute of Innovative Medical Equipment, United Imaging, Shenzhen, Guangdong China

**Keywords:** Cancer, 18F-fluorodeoxyglucose, Positron emission tomography, Total-body imaging

## Abstract

**Objectives:**

Commercialized total-body PET scanners can provide high-quality images due to its ultra-high sensitivity. We compared the dynamic, regular static, and delayed 18F-fluorodeoxyglucose (FDG) scans to detect lesions in oncologic patients on a total-body PET/CT scanner.

**Materials & methods:**

In all, 45 patients were scanned continuously for the first 60 min, followed by a delayed acquisition. FDG metabolic rate was calculated from dynamic data using full compartmental modeling, whereas regular static and delayed SUV images were obtained approximately 60- and 145-min post-injection, respectively. The retention index was computed from static and delayed measures for all lesions. Pearson’s correlation and Kruskal–Wallis tests were used to compare parameters.

**Results:**

The number of lesions was largely identical between the three protocols, except MRFDG and delayed images on total-body PET only detected 4 and 2 more lesions, respectively (85 total). FDG metabolic rate (MRFDG) image-derived contrast-to-noise ratio and target-to-background ratio were significantly higher than those from static standardized uptake value (SUV) images (*P* < 0.01), but this is not the case for the delayed images (*P* > 0.05). Dynamic protocol did not significantly differentiate between benign and malignant lesions just like regular SUV, delayed SUV, and retention index.

**Conclusion:**

The potential quantitative advantages of dynamic imaging may not improve lesion detection and differential diagnosis significantly on a total-body PET/CT scanner. The same conclusion applied to delayed imaging. This suggested the added benefits of complex imaging protocols must be weighed against the complex implementation in the future.

**Clinical relevance:**

Total-body PET/CT was known to significantly improve the PET image quality due to its ultra-high sensitivity. However, whether the dynamic and delay imaging on total-body scanner could show additional clinical benefits is largely unknown. Head-to-head comparison between two protocols is relevant to oncological management.

**Supplementary Information:**

The online version contains supplementary material available at 10.1186/s40644-023-00649-5.

## Introduction

Whole-body 18F-fluorodeoxyglucose (FDG) PET/ CT is often acquired at a specified time point, usually 60 min following the tracer injection [1]. FDG uptake is studied either qualitatively or semi-quantitatively using the standardized uptake value (SUV) [2]. This practice is generally acceptable for both reproducibility and repeatability. However, lesion SUV has a fundamental limitation as it represents a combination with FDG-6-phosphate (FDG-6-P) retained in the targeted tissue and the unbound FDG in the background [3]. This could restrict the accurate qualitative assessment of lesions in the organs or tissues with a high background uptake. Furthermore, SUV may not be the best option for applications requiring accurate measurement of specific FDG uptake, such as treatment response assessment [4].

Delayed acquisition (e.g., 90-min post-injection) can result in PET images with reduced background activity and possibly increased lesion activity, thereby generating a higher target-to-background ratio (TBR) than the regular protocol [5]. However, it requires prolonged scan time and provides low-count statistics. Nonetheless, this limitation can be outweighed by advantages in certain clinical scenarios. Delayed FDG PET/CT imaging of gliomas has been reported to efficiently distinguish between tumor and gray matter [6]. In addition, it can differentiate recurrence following radio-chemotherapy inflammation or a scar [7]. It is also beneficial for hepatocellular carcinoma and pancreatic tumors [8, 9]. Delayed imaging has certain limitations; for instance, it requires repositioning for the second PET/CT acquisition, requiring additional CT radiation. Moreover, 18-F decay may result in high noise, resulting in insufficient detected photon counts for reconstructing an image with acceptable quality. The advantage of high lesion contrast may therefore be offset, especially for less-avid lesions.

Dynamic FDG imaging increases lesion conspicuity by providing additional parametric images based on mathematical modelling other than just one “lumped” image. Standard dynamic imaging requires more than 60 min list-mode acquisition initiating with a tracer injection [10]. This facilitates the quantification of biological processes, such as metabolism or receptor binding via specific radiotracers based on kinetic modelling. For FDG, most existing studies are based on Patlak graphical analysis [11], provides the net FDG influx rate (K_i_) or metabolic rate (MRFDG) into tissues, thereby extracting the bound FDG-6-P signal from the entire uptake. Such differentiation enables lower background activity and hence higher TBR that facilitates lesion detection [12–17]. A full dynamic acquisition initiated from the injection can further involve micro-kinetic parameters, e.g., K_1_, k_2_, k_3_, etc., which have been proven to be effective in disease staging and treatment assessment [14, 18–20]. Similar to delayed imaging, dynamic FDG imaging has certain limitations. First, its implementation is complicated due to the prolonged acquisition. Second, it can be difficult to obtain the image-derived input function (IDIF) with no large artery in the field-of-view (FOV). IDIF derived from a small artery (e.g., carotid) can be underestimated [21, 22]. Third, densely reconstructed frames with enough detected counts at an early scan may be required to obtain multiple parametric images and capture tracer kinetics [23].

The introduction of the commercialized total-body scanners, i.e., uEXPLORER [24] and Biograph Vision Quadra [25], has allowed long coverage of human body. Ultra-high sensitivity facilitates both dynamic and delayed imaging with high image quality, which is beneficial in addressing the low count. Furthermore, IDIF can be extracted directly from large arteries such as the descending aorta. Micro- and macro-parameters at all critical organs can then be obtained. The enhanced quality of dynamic images allows better estimation of the whole-body parametric images [26, 27].

In this work, we performed both dynamic and delayed imaging in oncologic patients on a total-body PET scanner. As stated earlier, both delayed and dynamic FDG-PET imaging distinguished the metabolic FDG from the background FDG. However, a head-to-head comparison of these two imaging techniques has not been conducted despite numerous previous separate investigations. Here, each patient was scanned continuously for the first 60 min, followed by a delayed acquisition. Three images, i.e., regular SUV, delayed SUV, and parametric MRFDG, were obtained for each patient. We first evaluated the lesion detectability and quantitative comparability. Next, we investigated whether the advantage of certain protocol could be converted into clinical value, i.e., improved lesion detection and better differential diagnosis.

## Materials and methods

### Patient demographics

**Fig. 1:**
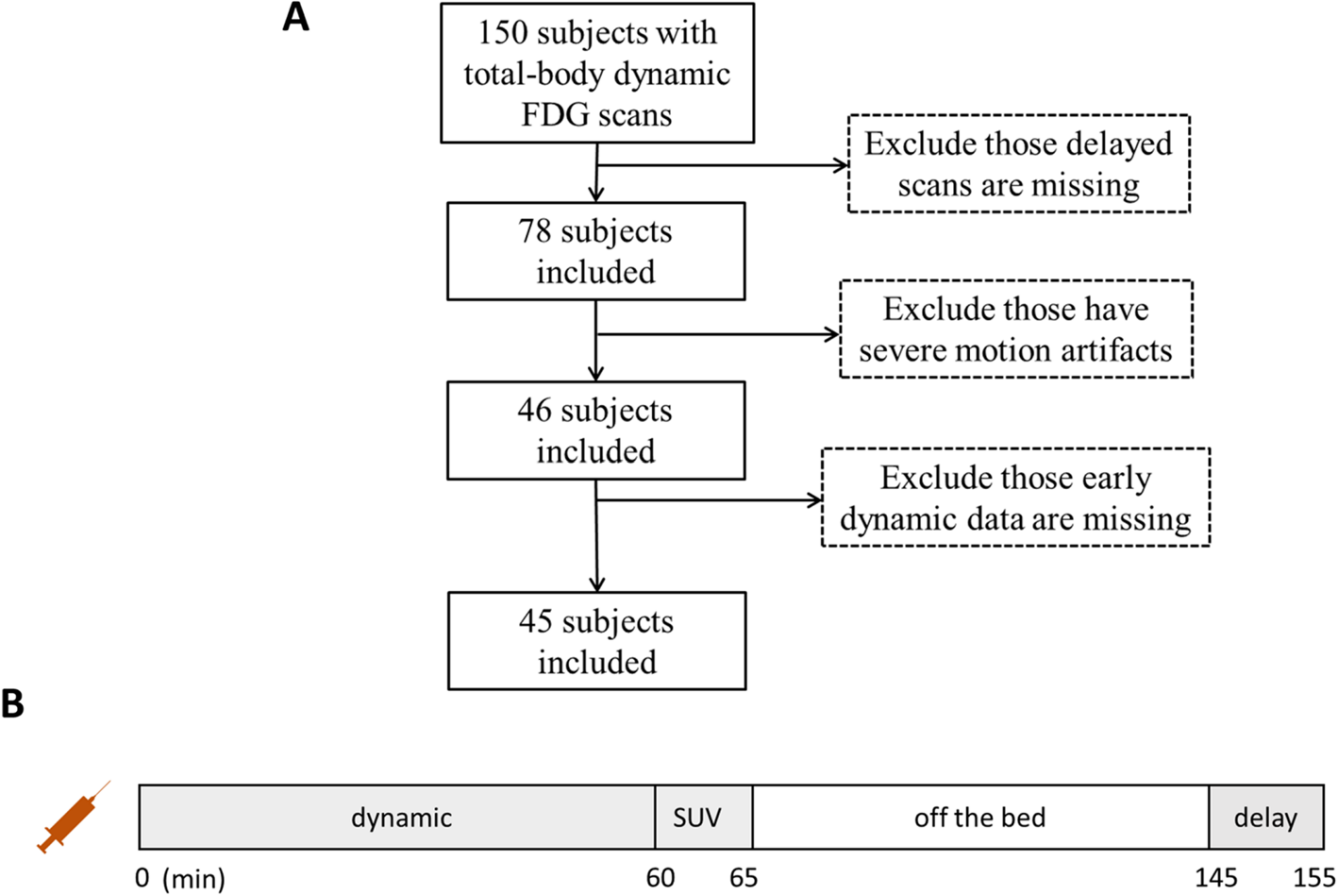
**A** Criteria for excluding scans. A total of 45 patients with lesions were studied retrospectively, including 21 patients with lung cancer, 6 patients with infection or inflammation, 13 patients with mediastinal lesions, and 5 patients with liver cancer as the primary suspicious lesion. **B** Scan protocol. A 65-min list-mode acquisition was initiated immediately after the bolus injection of FDG on a uEXPLORER PET/CT scanner. The acquired data were binned into 67 frames (5 s × 24, 10 s × 6, 30 s × 6, 60 s × 6, 120 s × 24, and 300 s × 1), for which the last frame was treated as a regular SUV image. The average start time of the delay scan was 145 min and lasted for 10 min

The study was approved by the local ethics committee. A total of 45 patients with tumor lesions in different locations were studied retrospectively for which the clinical indication for PET/CT imaging was staging of suspected malignancy. The inclusion criteria are shown in Fig. 1A. Each patient underwent both dynamic and delayed FDG scans on a uEXPLORER PET/CT scanner (United Imaging Healthcare, Shanghai, China) from December 2020 to July 2021 at the Henan Provincial People’s Hospital. Detailed information (age, sex, weight, injection dose, and suspicious lesion type/location) is listed in Table 1.

**Table 1:** Patient population in the current retrospective study^a^, with primary suspicious lesion type, age, gender, weight, and dose administration

Primary suspicious lesion type	Age (y/o.)	Gender (F:M)	Weight (kg)	Dose administrated (MBq)
Lung tumor (21)	58.7 ± 9.4	12:9	62.3 ± 10.9	250 ± 43
Mediastinal lesion (13)	53.2 ± 7.6	5:8	71.1 ± 9.8	281 ± 59
Liver and others (5)	52.7 ± 8.2	2:3	70.8 ± 6.9	282 ± 45
Infection and Inflammation (6)	52.1 ± 8.5	3:3	69.1 ± 6.2	277 ± 41
**Total** (45)	55.6 ± 8.5	25:20	66.7 ± 9.5	266 ± 46

^a^All exams were conducted for staging purpose in lung/liver

### Scan protocol

The complete scanning protocol is depicted in Fig. 1B. First, a CT scan was performed for attenuation correction. Next, a 60-min PET list-mode acquisition was initiated with a bolus injection of the tracer via the ankle vein. List-mode data were binned into 67 frames (5 s × 24, 10 s × 6, 30 s × 6, 60 s × 6, 120 s × 24, and 300 s × 1) and reconstructed into 192 × 192 × 673 matrices with voxel size of 3.125 × 3.125 × 2.866 mm^3^ using the 3D-ordered subset expectation maximization (OSEM) (PSF-TOF, 2 iterations, and 28 subsets). The last frame of the image (5 min) was converted into SUV value by normalizing it to the patient’s weight and injection dose and treated as a regular SUV image (SUV60). After initial imaging acquisition, the patient left the scanner and waited for a delayed scan. A second PET/CT scan was initiated 120 to 180 (154 ± 12) min post-injection for each subject and lasted for 10 min. Finally, the delayed SUV image was reconstructed with the same parameters as a regular SUV image, followed by a 2-mm Gaussian post-smoothing.

### Dynamic image processing

For dynamic imaging, voxel-based kinetic modelling was performed to generate parametric images. FDG is assumed to follow the irreversible two-tissue three-compartment model (irreversible two-tissue three-compartment [2T3k]). Considering the large number of voxels, the non-linear estimation problem was reformed into a linearized problem [28]. Afterward, the least squares algorithm was applied to solve parameters K_i_ (mL/g/min) and distribution volume (DV) at each voxel. K_i_ image was transformed to an MRFDG image (µmoL/g/min) by multiplying the blood glucose levels measured before the scan. IDIF was extracted from the ascending aorta by drawing a 10-mm-diameter region-of-interest (ROI) on six consecutive slices in an image obtained by summing early frames (0–60 s [29]). The delay (arrival time) between the body tissue and the aorta was computed using the leading-edge method [30]. Time–activity curve (TAC) at each voxel was aligned to the input function by selecting 10% of the peak value of the first 120 s as the trigger threshold to mark the arrival time. Neither the difference in uptake between blood and plasma nor the dispersion accounted for correction.

### Lesion detection

All target lesions were identified by two experienced nuclear medicine physicians (with 6 and 8 years of experience) who were blinded to the clinical information. The lesions were categorized into benign, indeterminate, and malignant lesions. The reference standard was based on the results from the follow-up surgery, biopsy, or complementary imaging techniques such as MRI or CT. In all, 73/85 lesions were confirmed by follow-up examinations (35 surgeries, 20 biopsies, and 18 imaging). The indeterminate lesions were determined if they missed the follow-up or could not be determined even after examination. Next, lesions were visualized and simultaneously detected in regular SUV, delayed SUV, and MRFDG images side-by-side on uWS-MI software version R004 (United Imaging Healthcare). In case physicians differed in their opinions, the final decision was made by consensus.

### Quantification comparison

All identified regions were delineated and analyzed. Each lesion was first delineated in the regular SUV image with a 50% cut-off threshold of the maximum intensity value by the physicians. Parametric MRFDG images were delineated to obtain the metabolic information at the same lesion. The lesions were re-delineated in the delayed images to calculate their corresponding mean SUV values, considering the potential positional mismatch between the first and second scans. All delineations were performed by the physicians using the software ITK-snap version 3.6.0. All computations below were performed with in-house MATLAB codes.

The mean TBR and CNR values for each lesion were computed for MRFDG, delayed, and regular images as a quantitative indication of lesion detectability. The mean TBR of a lesion was calculated as follows [17],

$$\mathrm{TBR }=\frac{\mathrm{Mean \,}({\text{lesion}})}{\mathrm{Mean \,}({\text{background}})}$$

This value was measured on each set of images. The background region was manually drawn as a spherical region for which the locations differed. For instance, for a lung lesion, the background region was drawn in the chest muscle. For the mediastinal lesion, it was drawn in the adjoint tissue in the mediastinum, and for a liver lesion, it was drawn in the background in the liver. The mean CNR of a lesion was defined by the lesion contrast divided by noise [17],

$$\mathrm{CNR }=\frac{{\text{Mean}\,}\left({\text{lesion}}\right)-{\text{Mean}\,}({\text{background}})}{{\upsigma }_{{\text{background}}}}$$

A higher TBR or CNR indicated better quantitative lesion detectability. The lesions were further categorized into lung lesions with high contrast and the others in the mediastinal and liver lesions with low contrast. The quantitative detectability of TBR and CNR was compared in each group.

Next, the relationship between the imaging protocols and the histology of lesions was evaluated. Specifically, we studied whether TBR and CNR could differentiate benign and malignant lesions. The retention index, with proved differential diagnostic value [31], was computed and compared as follows:

$$\mathrm{Retention \,index }\left({\text{RI}}\right)=\frac{{{\text{Mean}\,}({\text{lesion}}}_{{\text{delay}}})-{{\text{Mean}\,}({\text{lesion}}}_{{\text{early}}})}{{{\text{Mean}\,}({\text{lesion}}}_{{\text{early}}})}$$

### Statistical analysis

All statistical analyses were performed using the Statistical and Machine Learning Toolbox in MATLAB version R2018b (Mathworks, Inc.). A threshold of 0.05 was considered significant. The Shapiro–Wilk normality test was used to determine whether the data showed a normal distribution. Normally distributed data are expressed as mean and standard deviation. Pearson’s correlation was used to assess the relationship between parameters from regular static, delayed, and MRFDG images. TBR and CNR between different categories were compared using the Kruskal–Wallis test among groups with the correction of multiple comparisons using Bonferroni’s method.

## Results

### Visual assessment

**Fig. 2:**
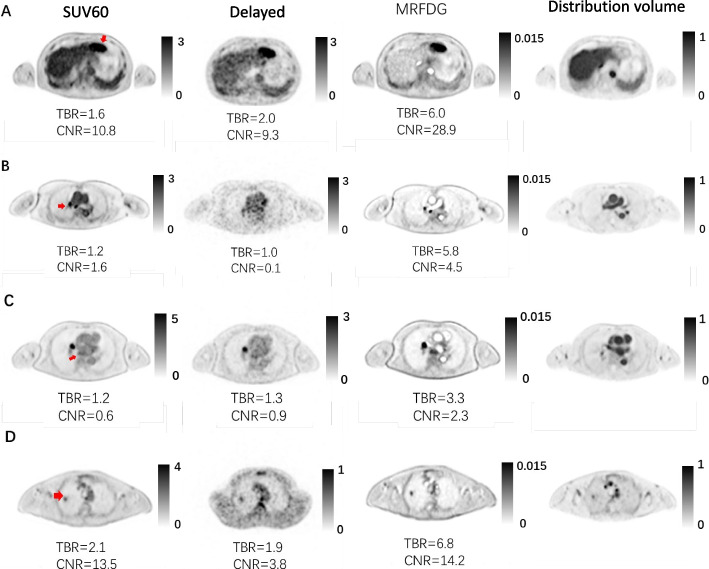
Cases with primary suspicious lesion type (**A**) liver cancer, (**B**) mediastinal lesion, and (**C**) lung cancer. The 60-min regular static images cannot confidently rule out the uptake foci (red arrows) from the background. They appear clear in delayed images and further in MRFDG images. For a lung lesion (**D**), the visual detectability among protocols was similar. Distribution volume images are shown in the last column

A total of 85 lesions were analyzed, as shown in Table 2, of which 22 were benign, 46 were malignant, and 17 were indeterminate. The most remarkable difference was the improved visual lesion detectability in the parametric MRFDG and delayed images compared to the regular images. An example is shown in Fig. 2A. This could be explained by the suppression of the blood compartment, particularly in the organs with a non-negligible blood component, such as the liver, spleen, and ventricles. For example, regular static images revealed FDG avid foci in soft tissues or adjoining vessels that could be dismissed as background (Fig. 2B, C). This is not observed in lung lesions due to the absence of the background activity surrounding the lesion (Fig. 2D). Delayed imaging was less superior to regular imaging because the alleviated noise level compromised the increased contrast. For example, in two lung tumors, two inflammation lesions, and one mediastinal lesion, the detectability was even inferior in the delayed imaging than in the regular acquisition (Fig. 2B).

**Table 2:** Lesion distribution with primary suspicious type, number of patients, diagnostic confirmation, and number of lesions

Primary suspicious lesion type	Nr. of patients	Dx	Nr. of ROIs	Total lesions
Lung lesion	21	benign	6	44
		indeterminate	10	
		malignant	28	
Mediastinal lesion	13	benign	7	25
		indeterminate	6	
		malignant	12	
Liver and others	5	benign	1	8
		indeterminate	1	
		malignant	6	
Infection and inflammation	6	benign	8	8
		indeterminate	0	
		malignant	0	
**Total**	45			85

**Fig. 3:**
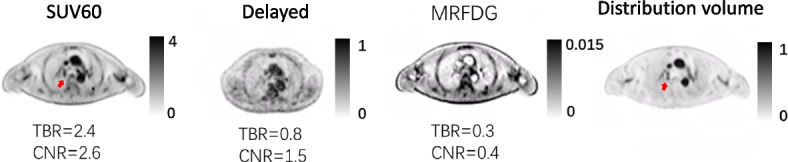
MRFDG and distribution volume static images excluded a suspicious lung lesion in a patient. It was visible in the SUV (red arrow) and distribution images but not in the MRFDG image, suggesting it to be a blood clot which was confirmed later

Despite the differences in reader confidence, the number of identified lesions was almost identical for MRFDG (85/85), delayed (83/85), and regular images (81/85). This suggests that the absence of background activity, e.g., in the liver or mediastinum facilitated the reading of MRFDG images, although only a few more pathological lesions were identified. In one patient with suspicious lung cancer, MRFDG excluded a suspicious lesion in an SUV image. Figure 3 shows the lesion in the SUV image, which is not visible in the MRFDG image. It was eventually confirmed as a benign blood clot, which was also observed in the DV image, reflecting the activity in blood volume.

### Quantitative comparison

**Fig. 4:**
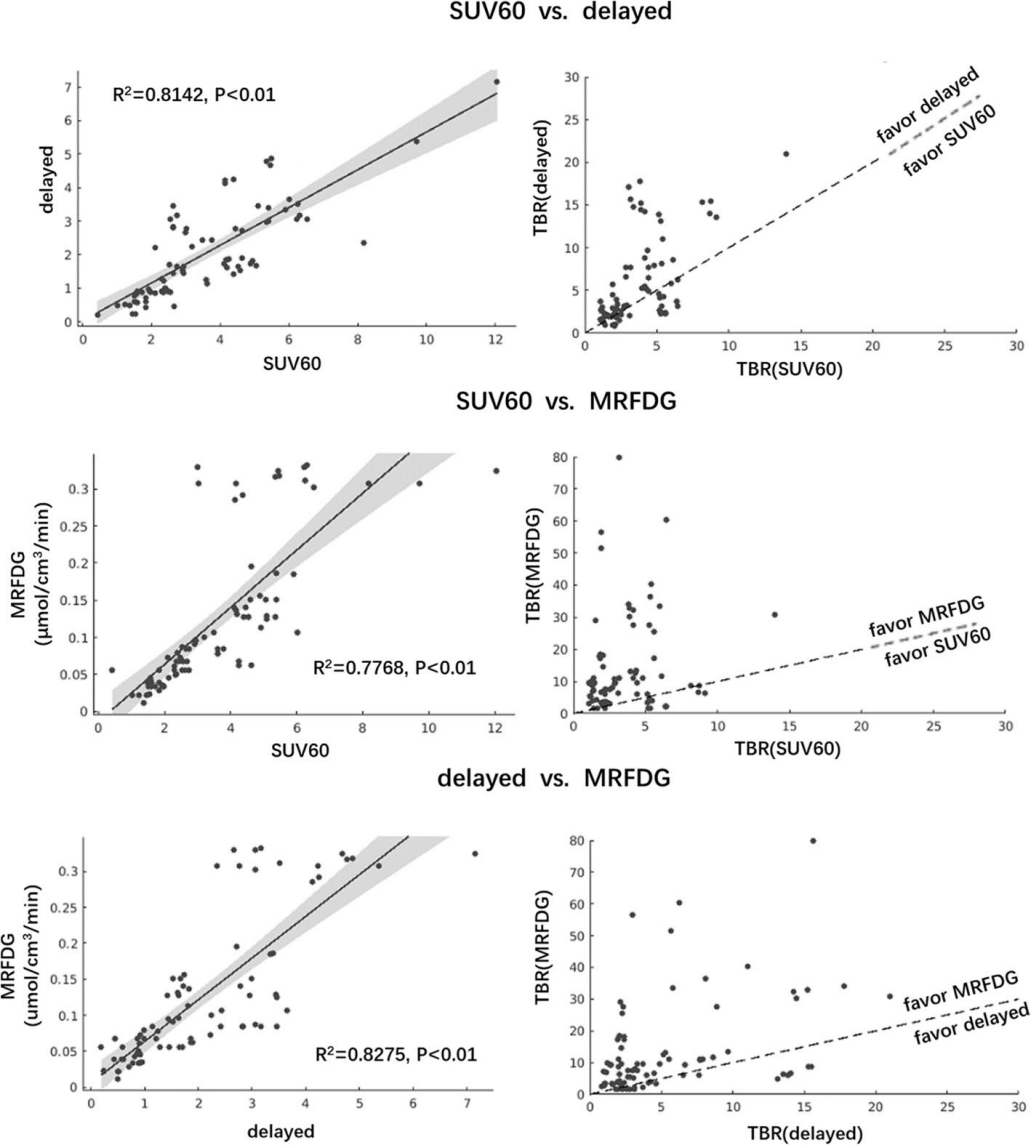
Left column: significant and paired correlations between regular SUV, delayed SUV, and quantitative MRFDG measures for all lesions (*P* < 0.01). Right column: corresponding TBR comparison

**Fig. 5:**
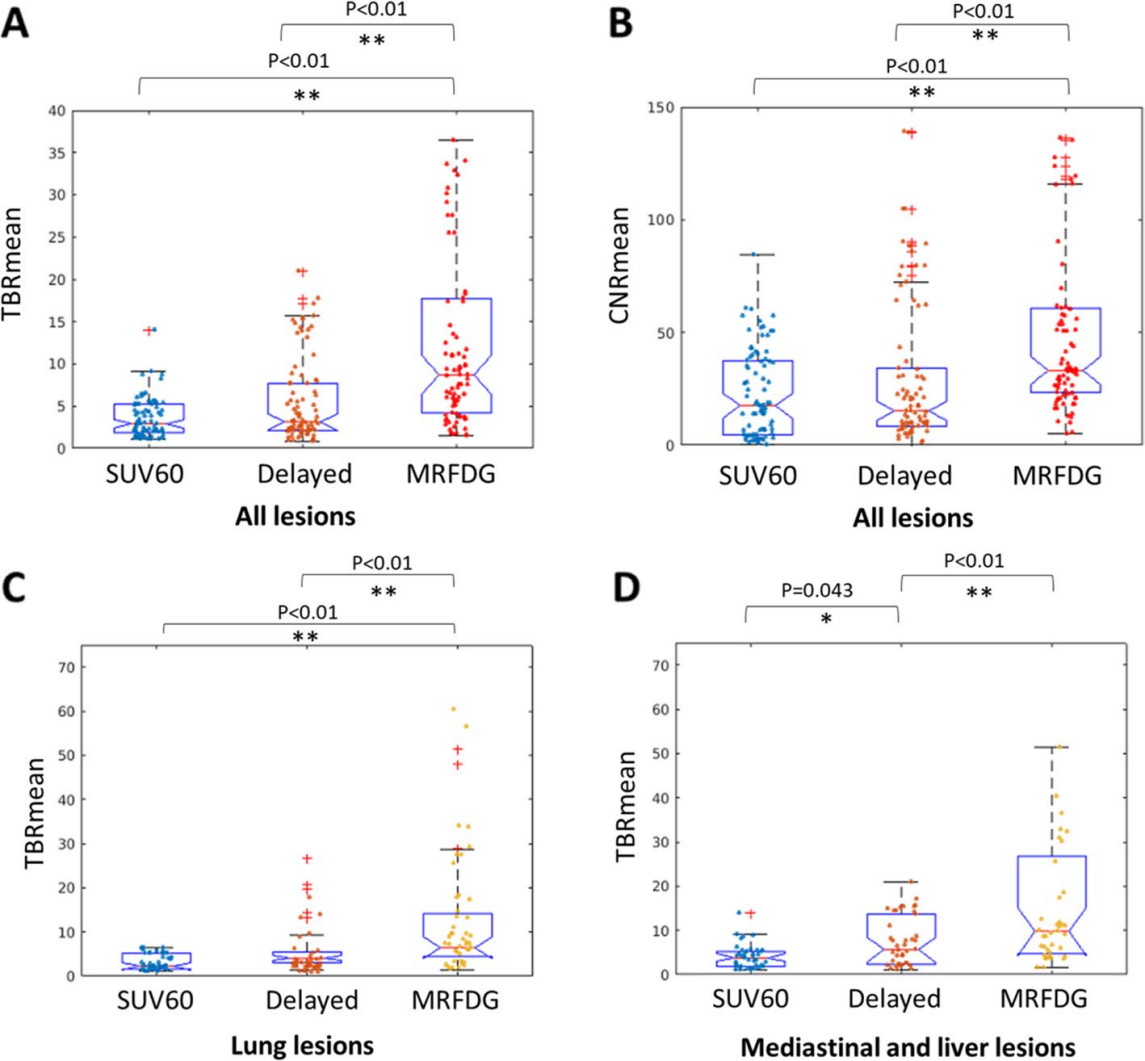
TBR (**A**) and CNR (**B**) values derived from MRFDG images were significantly higher than those obtained from static images (3.56 ± 2.93 vs. 15.29 ± 17.96 for TBR, *P* < 0.01, 22.72 ± 19.29 vs. 56.29 ± 51.53 for CNR, *P* < 0.01). However, this was not the case when comparing delayed images with regular static images (3.56 ± 2.93 vs. 5.52 ± 4.95, *P* = 0.078 for TBR; 22.72 ± 19.29 vs. 20.72 ± 36.55, *P* = 0.59 for CNR). TBR was further assessed after dividing lesions into (**C**) pulmonary and (**D**) non-pulmonary lesions. Pulmonary lesions showed a considerably low background signal, for which TBR in the delayed image was not significantly different (*P* = 0.682, Fig. 5C). However, it was significantly different for the tumors in mediastinal and liver regions (*P* = 0.043, Fig. 5D). In contrast, TBR from the MRFDG was significantly higher in either group of lesions (*P* < 0.01)

Lesion TBRs and CNRs for each protocol were compared. Pearson’s correlation analysis revealed that regular SUV, delayed SUV, and MRFDG values of lesions correlated with each other (paired *R*^2^ = 0.81, *R*^2^_=_ 0.78, *R*^2^_=_ 0.83, all *P* < 0.01, Fig. 4). The scatter plots in Fig. 4 display the comparison of their derived mean TBRs. Specifically, TBR derived from MRFDG images was significantly higher than that derived from regular static images (3.56 ± 2.93 vs. 15.29 ± 17.96, *P* < 0.01, Fig. 5A), with seven exceptional cases that were confirmed as benign. Similarly, MRFDG image-derived CNR was significantly higher than that obtained from regular images (22.72 ± 19.29 vs. 56.29 ± 51.53, *P* < 0.01, Fig. 5B).

On the other hand, this is not the case when comparing regular static and delayed images (3.56 ± 2.93 vs. 5.52 ± 4.95, *P* = 0.078 for TBR; 22.72 ± 19.29 vs. 20.72 ± 36.55, *P* = 0.59 for CNR). In this study, although the activity in blood pool significantly declined with time, it did not consistently translate into improved TBRs. For example, pulmonary lesions had a considerably low background signal, for which TBR in the delayed image was not significantly different (*P* = 0.682, Fig. 5C). They were significantly different for tumors in mediastinal and liver regions (*P* = 0.043, Fig. 5D). In contrast, TBR derived from MRFDG images was significantly higher in either group of lesions (*P* < 0.01). A similar trend was observed for CNR, except that delayed imaging was further restricted by high noise/low counts (Supplement Fig. 1).

### Differential diagnosis

**Fig. 6:**
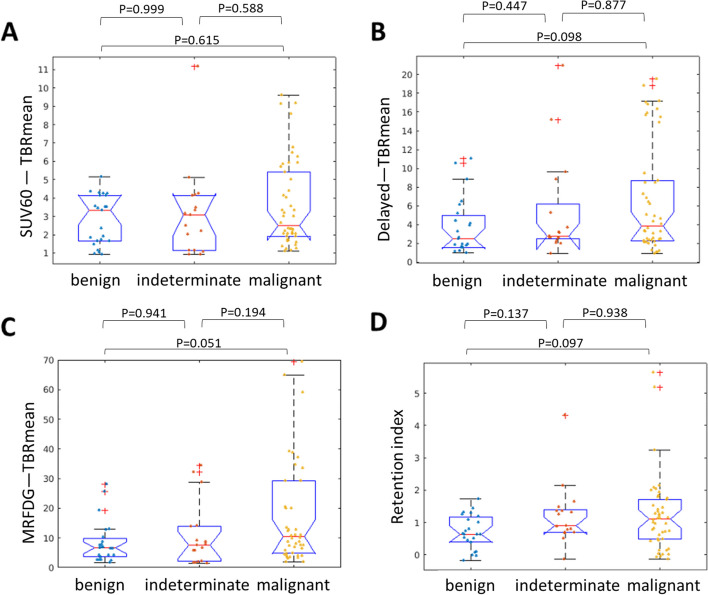
MRFDG exhibited a better performance in distinguishing benign and malignant lesions (**C**, *P* = 0.051, effect size 0.615) than the other two measures and (**A**, *P* = 0.588, effect size 0.389 for regular static image; **B**, *P* = 0.098, effect size 0.563 for delayed image). The retention index was calculated from regular and delayed static images (**D**), which was also inferior to MRFDG. However, all measures could not significantly differentiate the lesion types

We evaluated whether each measure could differentiate benign and malignant lesions based on follow-up confirmation of the pathology. Figure 6 shows the differential capability between quantitative (MRFDG) and semi-quantitative (regular and delayed SUV) measurements. None of these could significantly distinguish between benign and malignant lesions. TBR calculated from MRFDG images performed better than that obtained from regular SUV at 60 min (*P* = 0.051, effect size 0.615 vs. *P* = 0.588, effect size 0.389) and from delayed imaging (*P* = 0.098, effect size 0.563). The retention index displayed inferior performance (*P* = 0.097, effect size 0.567) to MRFDG. Similar trends were noted for CNR (Supplement Fig. 2).

## Discussion

To the best of our knowledge, this study is the first one to conduct a head-to-head comparison of FDG dynamic and delayed imaging for oncologic applications. Total-body scanner was known to have ultra-high sensitivity that can provide better image quality than conventional PET/CT scanner [24, 29, 32, 33]. In addition, dynamic PET imaging was previously restricted to single-bed positions and time-consuming invasive blood sampling. Total-body coverage would permit obtaining non-invasive input functions from the aortic arteries close to the arterials sampled [34, 35]. We assessed lesion detectability, quantification, and classification accuracy in 45 patients who underwent a total-body PET scan, with three scan modes: regular static imaging, delayed imaging, and dynamic imaging. It would be interesting to investigate whether the findings of this study could translate to a scanner with a regular axial FOV.

The measured values from different protocols correlated well (Fig. 4), as evident from the overall number of identified lesions that remained almost identical for regular SUV, delayed SUV, and dynamic MRFDG images, with 4 exceptions of 85 in favor of MRFDG. In certain cases, the DV image was used to exclude a possible lesion (Fig. 3). When it comes to quantification, MRFDG images had significantly higher CNR and TBR quantitative ability than regular static images, whereas delayed images did not always show this capacity, especially for lung lesions that were devoid from the background (Fig. 5C). These results are consistent with different visual detectability results obtained in parametric MRFDG and delayed images. On the other hand, the quantitative superiority of MRFDG did not necessarily result in significant differentiation between benign and malignant lesions (Fig. 6). However, a trade-off between the complexity of implementation (see Introduction) and accurately representing the FDG kinetics should be carefully considered.

For delayed imaging, higher TBR and CNR did not necessarily result in improved lesion detectability, which could contradict previously reported findings [5, 36]. This can partially be explained as most patients in this study suffered from pulmonary diseases, where the pathological lesions were primarily located in a tissue (liver) devoid of background activity. Therefore, comparable lesion detectability was predictable among protocols, although delayed imaging could increase the diagnostic accuracy of a liver or mediastinal region because normal tissues in these organs exhibit a high uptake (Fig. 5D). A similar observation was noted for the dynamic FDG imaging as MRFDG is useful for high-uptake lesions surrounded by a high background activity [37]. The possibility that MRFDG will offer additional insights into low-uptake tumors with low background activity is less. For a similar reason, diabetic individuals with poor glucose clearance would benefit from dynamic and delayed imaging as they have a high background uptake.

Several practical distinctions exist between dynamic and delayed imaging. First is the scan time. Dynamic imaging begins immediately after the tracer injection and lasts over 60 min, whereas delayed imaging initiates at least after 90 min. Second, dynamic imaging quantifies the net influx rate, together with micro-parameters, which cannot be achieved by semi-quantitative delayed imaging. Third, the major advantage of delayed imaging is its increased sensitivity in lesion detection, whereas kinetical information in dynamic imaging can further increase the specificity [20, 38, 39]. Lastly, there is no generally accepted delayed scan time point [40], and the parametric information derived from dynamic imaging is less time-dependent [41].

The current study has certain limitations. First, only a limited number of patients were involved because it is challenging to identify and recruit patients undergoing both dynamic and delayed imaging. It is necessary to include a population with different types of cancer patients. A related issue is that motion artefacts could affect the quality of regular, delayed, and MRFDG images to different degrees, thereby preventing scan inclusion (45/78 in this study). The movement should be corrected whenever possible [42, 43]. Second, we selected datasets retrospectively, for which the interval between initial and delayed acquisition varied between 120 and 180 min. Ideally, the same delayed interval should be applied. Third, tissues such as the liver, show reversible kinetics with a high rate of glucose dephosphorylation, which could result in biased MRFDG values [44]. Thus, a more appropriate tissue-specific model is warranted. Fourth, the slope of the linear regression can be calculated from the Patlak analysis as a surrogate of MRFDG [11]. Patlak analysis is a graphical analysis technique that derives from the full compartment model. Compared with full modelling, Patlak analysis requires the data after equilibrium and hence requires less scan time if a reliable population-based input function is available. Future studies should focus on their comparison.

## Conclusion

We conducted a head-to-head comparison of delayed and dynamic FDG protocols on a total-body PET scanner, to detect and differentially analyze the lesions in cancer patients. On a total-body PET scanner, the dynamic protocol provided quantitative advantages over delayed SUV measure, especially for lesions in tissues with significant background (e.g., blood abundant organs). On the other hand, it could not offer an obvious advantage in lesion detection and differential diagnosis when compared to regular static SUV measure. Moreover, a dynamic or delayed imaging protocol consists of more laborious procedures than a regular protocol. Altogether, trade-off between the complexity of implementation and accurately representing the FDG kinetics should be carefully considered while applying these protocols.

### Supplementary Information

**Additional file1: Supplement Figure 1.** CNR were compared by dividing the lesions into the lung (A) and non-lung ones (B). In both regions, CNR from the delayed image was not significantly different from the ones of the regular SUV(60) (*P*=0.139, *P*=0.769). CNR from the MRFDG, on the other hand, was significantly higher in either group of lesions (*P*<0.01). **Supplement Figure 2.** When quantifying CNR values, MRFDG has a better performance two (*P*=0.068, effect size 0.601) than the other two (*P*=0.963, effect size 0.103 for regular static image; *P*=0.162, effect size 0.495 for delayed image) in distinguishing benign and malignant lesions. Although, all measures cannot differ the lesion types significantly.

## Data Availability

The datasets supporting the conclusions of this article are available upon reasonable request.

## Abbreviations

SUV: Standardized uptake value
FOV: Field-of-view
FDG: Fluorodeoxyglucose
IDIF: Image-derived input function
GLUT: Glucose transporter
ROI: Region-of-interest
MRFDG: FDG metabolic rate
OSEM: Ordered subset expectation maximization
DV: Distribution volume
PSF-TOF: Point spread function, time-of-flight
TBR: Target-to-background ratio
2T3k: Irreversible two-tissue three-compartment model
CNR: Contrast-to-noise ratio
TAC: Time-activity curve
