# Supplementary material for: The role of dynamic, static, and delayed total-body PET imaging in the detection and differential diagnosis of oncological lesions
Source: Cancer Imaging. 2024 Jan 2;24:2. doi: 10.1186/s40644-023-00649-5 (PMC10759379; doi:10.1186/s40644-023-00649-5)
Supplement: Supplementary file 1 — Additional file1: Supplement Figure 1. CNR were compared by dividing the lesions into the lung (A) and non-lung ones (B). In both regions, CNR from the delayed image was not significantly different from the ones of the regular SUV(60) (P=0.139, P=0.769). CNR from the MRFDG, on the other hand, was significantly higher in either group of lesions (P<0.01). Supplement Figure 2. When quantifying CNR values, MRFDG has a better performance two (P=0.068, effect size 0.601) than the other two (P=0.963, effect size 0.103 for regular static image; P=0.162, effect size 0.495 for delayed image) in distinguishing benign and malignant lesions. Although, all measures cannot differ the lesion types significantly. [file 40644_2023_649_MOESM1_ESM.docx]

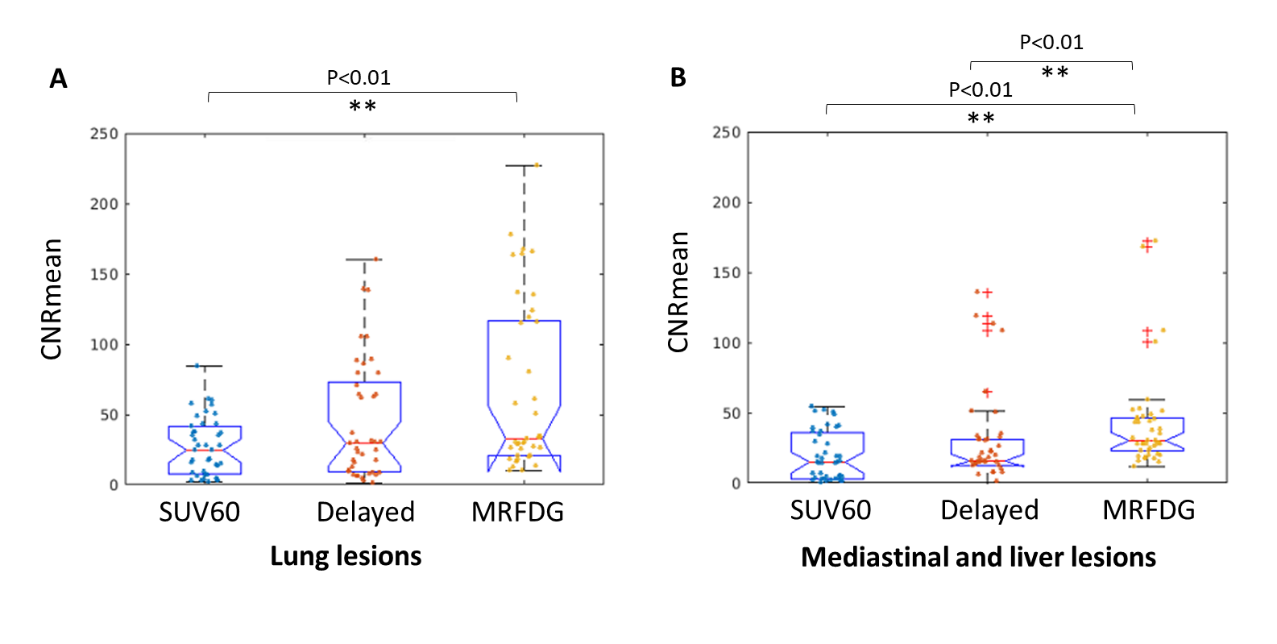


**Supplement Figure 1** CNR were compared by dividing the lesions into the lung (A) and non-lung ones (B). In both regions, CNR from the delayed image was not significantly different from the ones of the regular SUV(60) (P=0.139, P=0.769). CNR from the MRFDG, on the other hand, was significantly higher in either group of lesions (P<0.01).


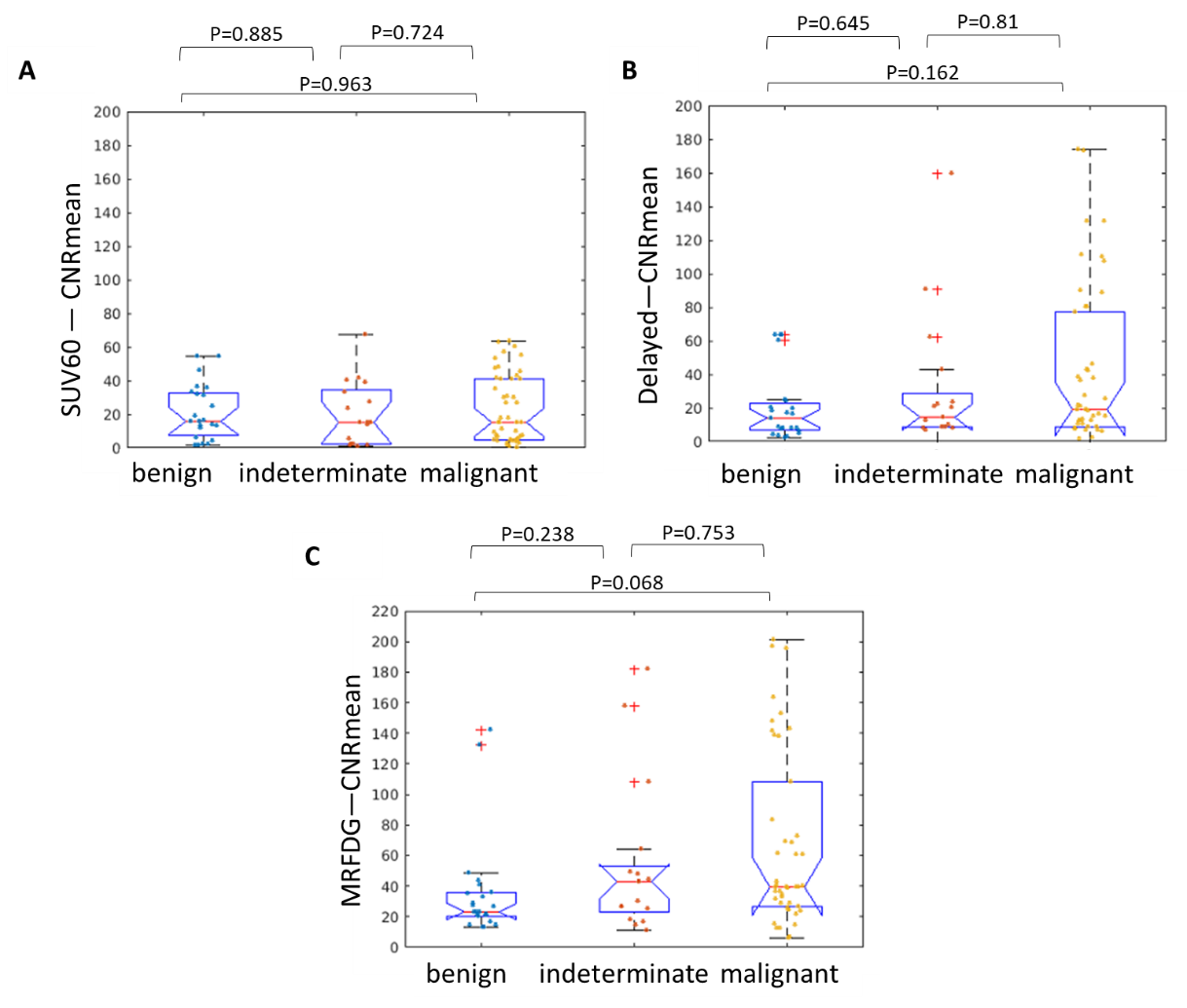


**Supplement Figure 2** When quantifying CNR values, MRFDG has a better performance two (P=0.068, effect size 0.601) than the other two (P=0.963, effect size 0.103 for regular static image; P=0.162, effect size 0.495 for delayed image) in distinguishing benign and malignant lesions. Although, all measures cannot differ the lesion types significantly.
